# Predicting Liver Disease Risk Using a Combination of Common Clinical Markers: A Screening Model from Routine Health Check-Up

**DOI:** 10.1155/2020/8460883

**Published:** 2020-05-31

**Authors:** Yi Wang, Yanni Li, Xiaoyi Wang, Ranko Gacesa, Jie Zhang, Lu Zhou, Bangmao Wang

**Affiliations:** ^1^Department of Gastroenterology and Hepatology, Tianjin Medical University General Hospital, Tianjin, China; ^2^Department of General Surgery, Beijing YouAn Hospital, Capital Medical University, Beijing, China; ^3^Department of Gastroenterology and Hepatology, University of Groningen and University Medical Center Groningen, Groningen, Netherlands; ^4^Department of Gastroenterology and Hepatology, People's Hospital of Hetian, Hetian, Xinjiang, China

## Abstract

**Background:**

Early detection is crucial for the prognosis of patients with autoimmune liver disease (AILD). Due to the relatively low incidence, developing screening tools for AILD remain a challenge.

**Aims:**

To analyze clinical characteristics of AILD patients at initial presentation and identify clinical markers, which could be useful for disease screening and early detection.

**Methods:**

We performed observational retrospective study and analyzed 581 AILD patients who were hospitalized in the gastroenterology department and 1000 healthy controls who were collected from health management center. Baseline characteristics at initial presentation were used to build regression models. The model was validated on an independent cohort of 56 patients with AILD and 100 patients with other liver disorders.

**Results:**

Asymptomatic AILD individuals identified by the health check-up are increased yearly (from 31.6% to 68.0%, *p* < 0.001). The cirrhotic rates at an initial presentation are decreased in the past 18 years (from 52.6% to 20.0%, *p* < 0.001). Eight indicators, which are common in the health check-up, are independent risk factors of AILD. Among them, abdominal lymph node enlargement (LN) positive is the most significant different (OR 8.85, 95% CI 2.73-28.69, *p* < 0.001). The combination of these indicators shows high predictive power (AUC = 0.98, sensitivity 89.0% and specificity 96.4%) for disease screening. Except two liver or cholangetic injury makers, the combination of AGE, GENDER, GLB, LN, concomitant extrahepatic autoimmune diseases, and familial history also shows a high predictive power for AILD in other liver disorders (*AUC* = 0.91).

**Conclusion:**

Screening for AILD with described parameters can detect AILD in routine health check-up early, effectively and economically. Eight variables in routine health check-up are associated with AILD and the combination of them shows good ability of identifying high-risk individuals.

## 1. Introduction

Autoimmune liver disease (AILD) is the second commonest cause of chronic liver disease in teenagers. There are several forms including autoimmune hepatitis (AIH), primary biliary cholangitis (PBC), primary sclerosing cholangitis (PSC), PBC-AIH, and PSC-AIH overlap syndromes (OS) which have common immunological characteristics and diagnosed based on immunological markers and histology [1–4]. AILD differs significantly in presentation and course depending on the patient's age at manifestation. Previous studies demonstrated that more than one-third of AILD patients had liver cirrhosis at the initial presentation, with the rate even being higher in PBC-AIH OS [5–8]. Therefore, it is necessary to develop a simple and reliable prognostic method for early identification of patients with high risk for AILD and help guide clinicians to identify potential AILD patients with maximized cost-effectiveness in primary and secondary healthcare systems.

It has been reported that AILD patients with cirrhosis at initial presentation have a substantially lower 10-year survival rate than patients without cirrhosis (61.9% vs. 94.0%) [9]. The prognosis and survival time of AILD patients largely depend on the development of liver cirrhosis and complications [10, 11]. Establishing practical methods for identifying high-risk individuals of AILD prior to the development of cirrhosis is crucial for improving the prognosis of patients with AILD.

In our previous study, we observed that abnormalities of several markers from routine health check-up, including serum biochemistry tests, family history of autoimmune diseases, and abdominal lymph node enlargement (LN) [12], might be helpful for predicting individuals at high risk. Other studies demonstrated that serum *γ*-globulins and abnormal LN ultrasound results were associated with AILD [13, 14]. Moreover, it is about 20-50% AILD patients have a history of other autoimmune diseases [15, 16], and 10-40% first-degree relatives of patients have autoimmune disorders [17, 18]. Nevertheless, there is a lack of evaluation of common clinical variables as the primary screening tool in clinical practice. For the aim of detecting AILD risk from routine health check-up, we analyze the clinical characteristics at initial presentation and select available indicators in health check-up. With these common indicators utilizing in the routine health check-up, we build up computational models for the prediction of AILD risk at the early clinical stage.

## 2. Materials and Methods

### 2.1. Study Design and Participants

This study was a retrospective long-term cohort study of 602 patients admitted to a single center from January 2001 to December 2017, including 173 patients with AIH, 330 with PBC, 78 with PBC-AIH OS, 19 with PSC, and 2 with Ig4 related liver disease. Informed written consent was obtained from all the study participants. All the patients were admitted fulfilling the diagnostic criteria of AILD, as proposed by diagnostic criteria of AIH (v.1999), PBC (v.2009), and “Paris Criteria” (v.1998) (Supplementary Materials—Participants (available here)). Additionally, 1000 individuals from the health management center were included as a healthy control group.

We recruited a cohort of individuals with abnormal liver function tests (LFTs) as validation cohort, which is including 56 AILD patients and 100 non-AILD liver disease cases with LFTs, including viral hepatitis, alcoholic liver disease, drug introduced liver injury, nonalcoholic fatty liver disease, and obscure liver injury (Supplementary Table 1). Both the AILD patients and non-AILD liver diseases were continuously diagnosed in 2018. The inclusion criteria, exclusion criteria, and the research design are shown in Supplementary Figure 1.

### 2.2. Data Collection

Demographic, clinical, laboratory, CT, and ultrasound imaging data of AILD patients were derived from the patient clinical records of Tianjin Medical University General Hospital (Supplementary Table 2). The data derived from the medical records in patients with AILD and healthy controls included age, gender, serum biochemical parameters (TP, ALB, GLB, ALT, AST, ALP, GGT, TBIL, and DBIL), LN, concomitant extrahepatic autoimmune diseases (CEAID), and familial history of autoimmune disease (FA). FA and CEAID were recorded via telephone follow-up interview, while cirrhosis was defined by CT image or liver biopsy, and LN were diagnosed by abdominal ultrasound [19]. FA was identified as at least one first-degree relative with at least one autoimmune disease, included AILD, autoimmune thyroid disease, Sjögren's syndrome, and rheumatoid arthritis. CEAID was defined as the patients were diagnosed with both AILD and extrahepatic autoimmune disease, the details were shown in Supplementary Table 3. To identify LN, the following criteria according to Soresi et al. were applied: one or more masses with an ovoid shape and less echogenic than the liver parenchyma, separated from adjacent organs and vessels by a clear-cut cleavage on repeated transverse, sagittal, and oblique scans [19]. Investigation sites included the area of the trunk of the portal vein, hepatic artery, celiac axis, superior mesenteric vein, and pancreas head. The ultrasound was performed by the same digestive specialist operator who was unaware of the clinical, biochemical, and histologic data. The study protocol adhered to the declaration of Helsinki and was approved by the Institutional Ethics Committee of Tianjin Medical University General Hospital.

### 2.3. Predictor Variables Selection

In order to select the AILD-associated variables for further analysis, we performed correlation analysis between the 14 indicators in the AILD cohort and retained noncorrelated variables such as age, gender, GLB, ALT, GGT, LN, CEAID, and FA for further analysis (Supplementary Table 4). We tested these variables for potential batch effects caused by the year of initial diagnosis. Univariate logistic regression analysis was used to affirm the association between each variable, and 8 variables were found to be significant and selected for the construction of AILD-risk models (Supplementary Materials—Choosing Variables). A comparison of variables between AILD patients and healthy controls is shown in Table 1.

### 2.4. Construction and Model Validation

After incomplete data filtering, we included 438 patients with AILD and 782 controls for model construction. All patients and controls were randomly split into training group (75% of data) and test group (remaining 25% of data). Models were trained using logistic regression and classification and regression trees (CART), with optimization performed by 3 repeats of 10-fold cross-validation on the training set. Model convergence and training were assessed using learning curves (Supplementary Figure 2). After establishing the first logistic regression model (Model 1) with 8 covariates, the two markers of liver and cholangetic injury (ALT and GGT) were subsequently excluded to better separate AILD patients and other abnormal LFTs cases. We trained logistic regression model (Model 2) and CART model with the remaining six variables (AGE, GEN, GLB, LN, CEAID, and FA). Details in the parameters of the CART model are provided in Supplementary Materials—Classification and Regression Tree [20].

The predictive power of models was calculated in the test group and the external validation group (56 cases with AILD and 100 controls with abnormal LFTs). The predictive power of the model was evaluated by receiver operating characteristic (ROC), area under the curve (AUC), accuracy, sensitivity, and specificity.

### 2.5. Statistical Analysis

We reported frequency (percentages) for categorical variables and median (range) for continuous variables. We used Chi-squared test and Mann-Whitney *U* test for comparisons of categorical and continuous variables, respectively. More details in statistical methods are described in Supplementary Materials—Descriptive analyses.

Correlation analyses and univariate logistic analyses were performed with SPSS (version 23.0, IBM, USA). Establishment and validation of the multivariate logistic regression model and CART model were performed in the R software (version 3.4.3.), using the caret package [21, 22]. Statistical tests were considered significant at *p* < 0.05.

## 3. Results

### 3.1. Study Cohort and Baseline Characteristics

We studied a total of 581 patients with AILD admitted to the hospital between January 2001 and December 2017, with three main subtypes: 173 AIH, 330 PBC, and 78 AIH-PBC OS. The number of newly diagnosed AILD patients increased yearly, from 3 cases in 2001 to 83 cases in 2017 (Supplementary Figure 3). The demographic and biochemical characteristics of the study population are outlined in Supplementary Table 2. The median age of all patients was 59 years (maximum 88 years and minimum 16 years), and the majority of the patients were female (86.2%). Overall, 242 out of 581 patients were asymptomatic (45%), and 191 patients had cirrhosis at first diagnosis (33%) of which 68 (35.6%) patients underwent liver biopsy. The characteristic of cirrhosis and noncirrhosis group was shown in Supplementary Table 5.

### 3.2. Changing of Detection Ways and Cirrhosis Rate at Diagnosis in AILD

AILD patients were classified into two groups due to admission reasons: the health check-up group referred to patients with abnormal LFTs or incidental findings detected in health check-up; and the symptomatic group included patients who had clinical symptoms, such as jaundice, gastrointestinal bleeding, and abdominal pain.

**Figure 1:**
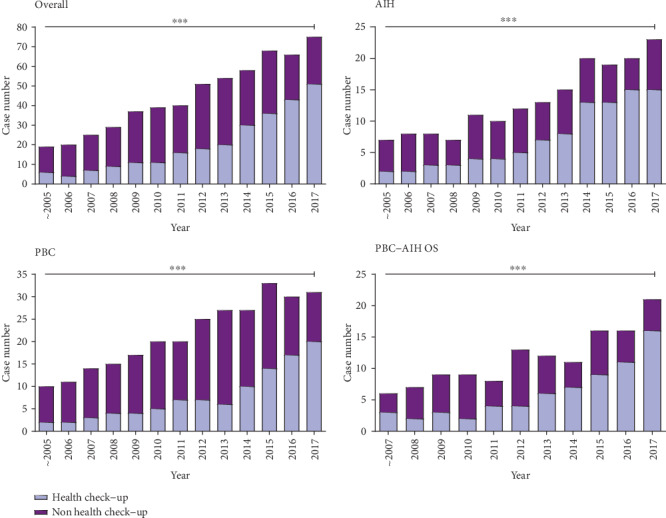
Proportion of AILD patients diagnosed through regular health check-up. The proportion increased year by year (all: *χ*2 = 44.32, *p* < 0.001; AIH: *χ*^2^ = 14.11, *p* < 0.001; PBC: *χ*^2^ = 18.97, *p* < 0.001; PBC-AIH OS: *χ*^2^ = 10.99, *p* < 0.001). *p* values were calculated by the Trend Chi-square test. ^∗∗∗^ represents *p* value lower than 0.001, ns. represents no statistically significance.

We found that the proportion of patients in the health check-up group increased from 31.6% before the year 2006 to 68.0% in the year 2017. This increase is statistically significant over the last 18 years (*χ*^2^ = 44.32, *p* < 0.001, Figure 1) and demonstrates that regular health check-up has become the key method to identify the individuals at high risk for AILD.

**Figure 2:**
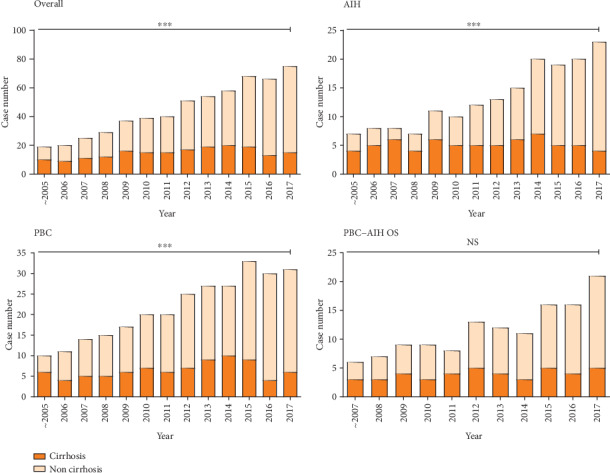
The rate of liver cirrhosis in AILD patients at initial admission to the hospital. Except for PBC-AIH OS patients, the proportion of liver cirrhosis was reduced year by year (all: *χ*^2^ = 19.36, *p* < 0.001; AIH: *χ*^2^ = 16.00, *p* < 0.001; PBC: *χ*^2^ = 6.95, *p* < 0.001; PBC-AIH OS: *χ*^2^ = 3.33, *p* > 0.05). *p* values were calculated by the Trend Chi-square test. ^∗∗∗^ represents *p* value lower than 0.001, ns. represents no statistically significance.

We further analyzed the rate of cirrhosis at diagnosis in AILD patients (Figure 2) and found that the proportion of patients with cirrhosis at baseline gradually decreased from 52.6% before the year 2006 to 20.0% in the year 2017 (*χ*^2^ = 19.36, *p* < 0.001). The trend of decrease was found in subgroups of AILD patients with AIH and PBC (*χ*^2^ = 16.00, *p* < 0.001; *χ*^2^ = 6.95, *p* = 0.008). The proportion of cirrhosis at baseline in patients with PBC-AIH OS showed a potential trend of decrease over time (*χ*^2^ = 3.33, *p* > 0.05).

### 3.3. Risk Factors of AILD in the Health Check-Up

Compared with healthy controls, 14 parameters measured during the routine health check-up were significantly associated with AILD (*p* < 0.001, Table 1). After pairwise correlation analysis, we excluded 6 parameters (TP, ALB, AST, ALP, TBIL, and DBIL) that were strongly correlated with others. Consequently, age, gender, GLB, ALT, GGT, LN, CEAID, and FA were assumed as independent variables and used to construct prediction models. The above eight variables were also found to be associated with AILD in univariate analysis (Table 2). The factor of positive abdominal lymph node enlargement showed the most significant association within them (OR 19.46, 95% CI 10.91-34.69, *p* < 0.001).

### 3.4. Development and Validation of Models for Prediction of AILD in Health Check-Up

**Figure 3:**
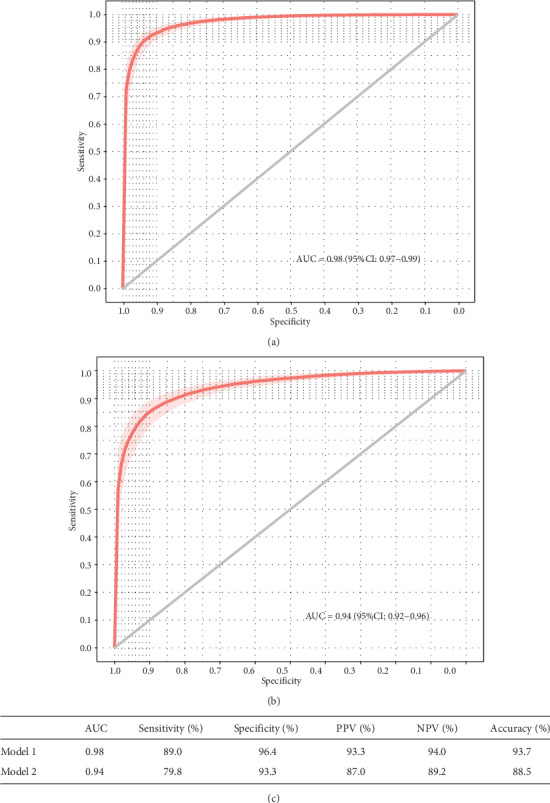
Performance of the logistic regression models in clinical parameters measured from routine health check-up. (a) Receiver operating characteristic curve (ROC) shows Model 1 had a good ability in predicting high-risk individuals with AILD in 8 variables, (b) Model 2 also showed good prediction ability without liver- or cholangetic injury parameters, (c) performance of two models in the test group. AUC: area under the receiver operating characteristic curve; PPV: positive predictive value; NPV: negative predictive value.

We build a logistic regression model for the identification of individuals at risk for AILD with healthy check-up participants in eight predictors (Model 1, Table 3) and evaluated this model using cross-validation. The model showed high predictive power for AILD in both the cross-validation set (Kappa = 0.87, accuracy = 0.94, Supplementary Figure 2A) and the test set composed of 25% of samples (AUC of 0.98; 95% CI 0.97-0.99, sensitivity of 89.0%, specificity of 96.4%, and accuracy of 93.7%, Figures 3(a) and 3(c)). The strongest predictor was positive abdominal lymph node enlargement (OR 8.85, 95% CI 2.73-28.69).

We constructed a logistic regression model without the variables of ALT and GGT, designed to separate AILD cases from patients with other hepatic or cholangetic diseases (Model 2, Table 3). This model showed high performance in cross-validation set (Kappa = 0.75, accuracy = 0.89, Supplementary Figure 2B) and the test set (AUC of 0.94; 95% CI 0.92-0.96, sensitivity of 79.8%, specificity of 93.3%, and accuracy of 88.5%, Figures 3(b) and 3(c)). Abdominal lymph node enlargement positive result (OR 17.24, 95% CI 7.18-41.41) was also found to be the most influential variable compared to others (Table 3).

**Figure 4:**
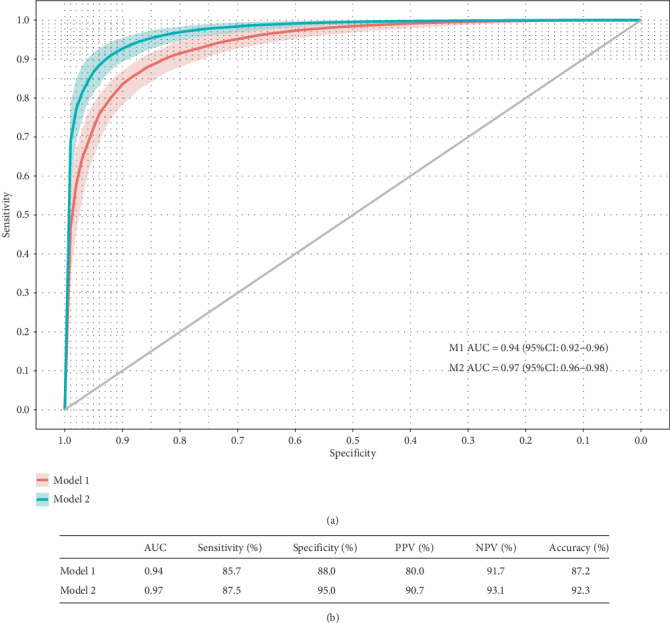
Performance of external validation cohort in other liver injuries with abnormal LFTs. Validating the two logistic models in external cohorts, (a) ROC shows a comparison of two models in the independent validation group, (b) performance of two models in the external validation group, which shows Model 2 with six variables has better ability to identify AILD from other liver diseases. ROC: receiver operating characteristic curve; AUC: area under the receiver operating characteristic curve; LFTs: liver function tests; PPV: positive predictive value; NPV: negative predictive value.

Next, we tested these two models in a newly collected cohort of 56 AILD patients and 100 individuals with other liver diseases. Here, model without liver biomarkers (Model 2) showed higher performance (AUC 0.97, 95% CI 0.96 to 0.98) when compared to Model 1 (AUC 0.94, 95% CI 0.92 to 0.96). The exclusion of the two liver biomarkers, which are not specific for AILD, increased both the sensitivity and specificity of AILD prediction (87.5% and 95.0%; Figure 4).

### 3.5. Decision Tree Model Simplifies Prediction of AILD with Health Check-Up Predictors

**Figure 5:**
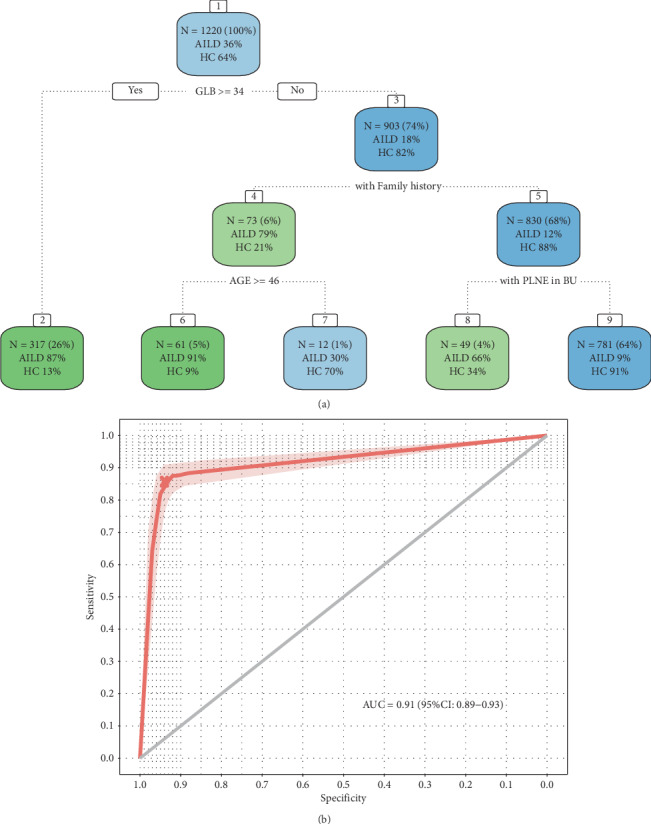
Classification trees (CART) for predicting high-risk individuals of AILD. (a) CART tree for prediction visualization. The two sets of numbers underneath each terminal node represent the proportions of patients or control subjects. The subgroups were marked with green and blue according to prediction outcomes, e.g., subgroup 2 represents the group with the predictive pattern for the disease (probability of AILD was 87%, dark green). The actual split values (thresholds) were indicated in the branches of the tree. HC: healthy controls; AILD: autoimmune liver disease patients. (b) Receiver Operating Characteristic Curve. The CART model worked well in predicting high-risk individuals with AILD in other liver injuries with an AUC of 0.91.

In order to find the best combination of predictors and their exact cutoff values, as well as establish a visualization prediction model, we fitted a CART model with six variables used for the training of Model 2. The fitted decision tree is shown in Figure 5(a), and the results of the evaluation on the external validation set are shown in Figure 5(b). The model demonstrated good predictive power for the identification of AILD cases (AUC, 0.91, 95% CI 0.89-0.93, sensitivity of 85.7%, specificity of 92.0%). Consistent with the logistic regression model, elevated GLB (≥34 g/L) was the most important discriminating factor between high and low-risk AILD, while increased age (>45 years), familial history of autoimmune disease and positive ultrasound finding of abdominal lymph node enlargement were also found to be important risk factors for AILD (Figure 5(a)).

## 4. Discussion

AILD is often asymptomatic at the early stage. Approximately 30% of patients have already developed cirrhosis when the disease has been diagnosed, and such patients have poor prognosis (e.g., lower survival rates). However, if patients with AILD can be identified and diagnosed prior to the onset of cirrhosis, treatments with immunosuppressive agents could significantly improve the survival rates (from 62% to 94%) [10, 23]. While the management of AILD is crucial, the early identification of the disease remains a challenge; currently no screening methods are available for identifying individuals at risk of AILD [2]. To the best of our knowledge, this is the first study that identified predictors measured in routine health check-up for the early detection of AILD.

In this study, we found that the proportion of cirrhosis in AILD patients gradually decreased over the past 20-year period (Figure 2). This is potentially because the increase in regular health check-up attendance allowed the identification of AILD patients with no clinical symptoms, but presented abnormal LFTs in the health check-up and were referred to a hepatologist for further diagnostic tests. This is in line with the study that investigated diagnostic rates of autoimmune hepatitis in Singapore, which concluded that the lack of awareness of the primary health care professionals and the public led to the delayed diagnosis and therapy of AIH [24]. Our study further suggests that regular health check-up may help improve early detection of individuals at high risk for AILD.

We found that 14 parameters measured in routine health check-up might contribute to the prediction models for AILD (Table 1). Of these, we chose 8 uncorrelated factors (AGE, GENDER, GLB, ALT, GGT, LN, CEAID, and FA) to build predictive models for identifying high-risk AILD patients. Among the transaminase and bile enzymes, AST and ALT, ALP and GGT are highly correlated. Previous researches showed that ALT and GGT are more “early” and “sensitive” indicators, which are more suitable for early screening than AST and ALP [25, 26]. Therefore, we finally chose ALT and GGT as the representative to enter the model (Supplementary Table 4). Using these variables, we developed two prediction models for the identification of high AILD risk: Model 1 is intended to be used in general health check-up for the identification of AILD risk with clinical variables, and we excluded LFTs in Model 2 to enable estimation of AILD risk in individuals with abnormal LFTs, to aim at identifying AILD from other liver diseases. While detection of abnormal LFTs in health check-up has a potential to identify AILD, it is not a specific marker because LFTs are elevated in different liver diseases [27, 28]. Thus, we used other parameters measured in the health check-up to design model for the specific identification of AILD.

The Model 1 is built up for general healthy check-up to identify high-risk AILD. Combined with the above clinical variables, we found high predictive power in the internal cross-validation (sensitivity is 89.0%, specificity is 96.4%, Figure 3). Model 2 showed higher specificity and higher sensitivity when tested using validation cohort of patients with AILD and other liver diseases (Figure 4). This implies that Model 2 without LFTs is better suited to the identification of AILD from different liver disorders manifest with abnormal LFTs. It is known that a family history of AILD and a history of other autoimmune diseases are risk factors for this disease [29], that AILD was found mainly in middle-aged women, and that serum *γ*-globulins and abnormal LN were associated with autoimmune hepatitis [2]. Among them, enlarged abdominal lymph nodes are a typical ultrasound feature, which is consistent with our results [30].

To demonstrate the possible implementation of our model in the clinical practice, we constructed a decision-tree based schematic for identification of AILD risk (CART model). This allowed us to quantify the cutoffs for selected variables and to assess the risk for subgroups (Figure 5). For example, the model predicts that individuals with GLB ≥ 34 g/L, older than 45 years, and with a family history of AILD are at a very high risk of AILD (risk > 90%) and should undergo further clinical tests for AILD diagnosis. While AILD is a female-dominant disease, gender was not identified to be a critical variable in our decision tree model (Figure 5(a)), possibly because it is mildly correlated with GLB in our data (Spearman correlation 0.33). For clinical practice, when an individual is judged to be “high risk” with abnormal LFTs, it is necessary to conduct the immunology or liver biopsy to further confirm the diagnosis of AILD, and it is also necessary to have virology, blood lipid, B-ultrasound, and other tests to estimate specific liver damages [31].

Since AILD is a rare disease (prevalence of 1-2 per 100,000 worldwide [32]), our models were, by necessity, designed using relatively small samples and an unbalanced ratio of cases and controls. Furthermore, while our model did show high performance in the external validation cohort, it might require further validation in cohorts from other medical centers. Finally, the predictive model was designed to supplement, rather than replace, the physician's clinical judgment and existing diagnostic criteria.

In summary, we demonstrate that models trained using limited sociodemographic and clinical parameters measured during a routine health check-up enable reliable identification of individuals at high risk for AILD. This approach could be implemented in both primary and secondary health-care settings to facilitate identification of noncirrhotic AILD patients at the early stage, and thus help improve the prognosis of patients with AILD.

## Figures and Tables

**Table 1:** Baseline characteristics of AILD cases and healthy controls.

Features	All patients (*n* = 581)	HC (*n* = 782)	Statistic value^∗^	*p* value
Gender(female/male)	501/80	397/385	143.09	<0.001
Age, y (range)	59 (16-88)	51 (38-60)	9.47	<0.001
Biochemical parameters				
TP (g/L)	76 (69-82)	74 (71-77)	3.01	0.003
ALB (g/L)	39 (34-43)	47 (45-48)	17.57	<0.001
GLB (g/L)	37 (32-42)	28 (25-30)	18.33	<0.001
ALT (IU/L)	73 (41-159)	17 (12-24)	20.69	<0.001
AST (IU/L)	76.9 (47-145)	18 (16-22)	23.02	<0.001
ALP (IU/L)	179 (115-328)	62 (52-74)	22.09	<0.001
GGT (IU/L)	191 (98-370)	21 (15-29)	21.92	<0.001
TBIL (*μ*mol/L)	19.6 (12.8-42.5)	10.9 (8.3-14.0)	14.50	<0.001
DBIL (*μ*mol/L)	7.6 (4.4-22.33)	4.0 (3.3-4.9)	13.46	<0.001
LN (%)	226 (38.9)	26 (3.3)	195.00	<0.001
CEAID (%)	257 (44.2)	39 (5.0)	167.19	<0.001
FA (%)	50 (8.6)	15 (1.9)	205.38	<0.001

^∗^Statistic value are calculated with the Chi-square test(*χ*^2^) in categorical variables and Mann-Whitney *U* test in numerical variables. Abbreviations: TP: total protein; ALB: albumin; GLB: globulin; ALT: alanine aminotransferase; AST: aspartate aminotransferase; ALP: alkaline phosphatase; GGT: gamma-glutamyl transpeptidase; TBIL: total bilirubin; DBIL: direct bilirubin; LN: abdominal lymph node enlargement (B-mode ultrasound); CEAID: current extrahepatic autoimmune diseases; FA: familial autoimmunity.

**Table 2:** Univariate regression analysis of factors measured by health check-up (*n* = 1220, 438 AILD cases vs. 782 healthy controls).

Factors	Log OR (*β*)	SE (*β*)	*p* value	OR	95% CI
Gender (female)	2.07	0.19	<0.001	7.90	5.43–11.49
Age at diagnosis (yrs)	0.07	0.01	<0.001	1.07	1.05–1.08
GLB (g/L)	0.30	0.02	<0.001	1.35	1.30–1.41
ALT (IU/L)	0.07	0.01	<0.001	1.07	1.06–1.08
GGT (IU/L)	0.03	0.002	<0.001	1.03	1.02–1.03
LN	2.97	0.30	<0.001	19.46	10.91–34.69
CEAID	2.05	0.20	<0.001	7.75	5.27–11.39
FA	2.80	0.28	<0.001	16.50	9.53–28.55

Log OR (*β*): logistic regression *β* coefficients (log odds ratio for one unit increase in the explanatory variable); SE (*β*): standard error for the *β* coefficient; OR: odds ratio for one unit increase in the explanatory variable (exponential of *β*); 95% CI: 95% confidence interval for the odds ratio; Abbreviations: AILD: autoimmune liver disease; GLB: globulin; ALT: alanine aminotransferase; GGT: *γ*-glutamyltransferase; LN: abdominal lymph node enlargement (B-mode ultrasound); CEAID: current extrahepatic autoimmune diseases; FA: familial autoimmunity.

**Table 3:** Logistic regression models with predictive variables measured by routine health check-up.

	Log OR (*β*)	SE (*β*)	*p* value	OR (exp[*β*])	95% CI OR
Model 1					
Gender (male as reference)	1.74	0.46	<0.001	5.69	2.29–14.12
Age at diagnosis (years)	0.09	0.02	<0.001	1.10	1.07–1.13
GLB (g/L)	0.25	0.04	<0.001	1.28	1.19–1.38
ALT (IU/L)	0.04	0.01	<0.001	1.04	1.03–1.06
GGT (IU/L)	0.01	0.002	<0.001	1.01	1.006–1.014
LN	2.18	0.60	<0.001	8.85	2.73–28.69
CEAID	1.26	0.47	0.007	3.51	1.41–8.76
FA	1.92	0.53	<0.001	6.85	2.41–19.49
Model 2					
Gender (male as reference)	1.53	0.33	<0.001	4.64	2.44–8.82
Age at diagnosis (years)	0.07	0.01	<0.001	1.07	1.05–1.10
GLB (g/L)	0.28	0.03	<0.001	1.32	1.26–1.39
LN	2.85	0.45	<0.001	17.24	7.18–41.41
CEAID	1.55	0.34	<0.001	4.72	2.41–9.23
FA	1.86	0.41	<0.001	6.41	2.84–14.44

Notes: Model 1 (M1) used the eight common variables after correlation analyses, and Model 2 (M2) used variables without liver- or cholangetic injury markers. Abbreviations: AILD: autoimmune liver disease; GLB: globulin; ALT: alanine aminotransferase; GGT: *γ*-glutamyltransferase; LN: abdominal lymph node. Enlargement (B-mode ultrasound); CEAID: current extrahepatic autoimmune diseases; FA: familial autoimmunity.

## Data Availability

The data used to support the findings of this study are available from the corresponding author upon request.

## Abbreviations

AILD: Autoimmune liver disease
AIH: Autoimmune hepatitis
PBC: Primary biliary cholangitis
PSC: Primary sclerosing cholangitis
OS: Overlap syndromes
LN: Abdominal lymph node enlargement
LFTs: Liver function tests
ALT: Alanine aminotransferase
GGT: *γ*-Glutamyltranspeptidase
TP: Total protein
ALB: Albumin
GLB: Globulin
AST: Aspartate aminotransferase
ALP: Alkaline phosphatase
TBIL: Total bilirubin
DBIL: Direct bilirubin
CEAID: Concomitant extrahepatic autoimmune diseases
FA: Familial autoimmunity
CART: Classification and regression tree
GEN: Gender
ROC: Receiver operating characteristic
AUC: Area under the curve
CIs: Confidence intervals
IgG: Immunoglobulin G
IgM: Immunoglobulin
ANA: Antinuclear antibody
AMA: Antimitochondrial antibody
AMA-M2: Antimitochondrial antibody-M2
SMA: Antismooth muscle antibody.

## Supplementary Materials

Supplementary Materials Supplementary Table 1: baseline demographic and clinical characteristics of other hepatic disease patients. Supplementary Table 2: baseline demographic and clinical characteristics of AILD patients. Supplementary Table 3: the percentage of CEAID from the study group. Supplementary Table 4: correlation matrix for the comparison between each predictive variable (*n* = 581). Supplementary Table 5: baseline demographic and clinical characteristics of cirrhosis patients and noncirrhosis patients. Supplementary Figure 1: flowchart of the inclusion criteria. Supplementary Figure 2: learning curve of regression model1 and model2. Supplementary Figure 3: the trend of newly diagnosed AILD patients yearly.
