# Supplementary material for: Predicting Liver Disease Risk Using a Combination of Common Clinical Markers: A Screening Model from Routine Health Check-Up
Source: Dis Markers. 2020 May 31;2020:8460883. doi: 10.1155/2020/8460883 (PMC7281844; doi:10.1155/2020/8460883)
Supplement: Supplementary Materials — Supplementary Table 1: baseline demographic and clinical characteristics of other hepatic disease patients. Supplementary Table 2: baseline demographic and clinical characteristics of AILD patients. Supplementary Table 3: the percentage of CEAID from the study group. Supplementary Table 4: correlation matrix for the comparison between each predictive variable (n = 581). Supplementary Table 5: baseline demographic and clinical characteristics of cirrhosis patients and noncirrhosis patients. Supplementary Figure 1: flowchart of the inclusion criteria. Supplementary Figure 2: learning curve of regression model1 and model2. Supplementary Figure 3: the trend of newly diagnosed AILD patients yearly. [file 8460883.f1.zip › Supplementary Tables_new.docx]

Supplementary Table 1: Baseline demographic and clinical characteristics of other hepatic disease patients.

| Features | Viral hepatitis  (n=34) | Alcoholic liver disease  (n=18) | Drug induced liver injury  (n=14) | NAFLD  (n=5) | Obscure liver injury  (n=29) |
| --- | --- | --- | --- | --- | --- |
| Gender (female/male) | 10/24 | 4/14 | 9/5 | 2/3 | 25/4 |
| Age females; y(range) | 54(36-63) | 53(42-61) | 58(38-65) | 56(30-63) | 59(41-70) |
| Biochemical Parameters |  |  |  |  |  |
| TP; g/L | 63(56-67) | 63(59-70) | 65(60-69) | 71(59-75) | 62(58-68) |
| ALB; g/L | 32(28-36) | 33(28-35) | 37(33-41) | 42(33-45) | 34(29-39) |
| GLB; g/L | 30(25-34) | 31(27-36) | 26(24-33) | 29(24-30) | 28(24-31) |
| ALT; IU/l | 32(19-99) | 27(19-35) | 113(26-257) | 57(23-183) | 31(15-69) |
| AST; IU/l | 42(27-80) | 35(25-64) | 55(29-98) | 41(35-58) | 28(20-46) |
| ALP; IU/l | 101(74-142) | 82(61-143) | 105(77-139) | 88(65-104) | 90(69-123) |
| GGT; IU/l | 54(29-127) | 141(42-486) | 127(52-247) | 84(41-336) | 70(39-173) |
| TBIL; umol/L | 24.3(15.7-49.6) | 18.9(12.0-35.9) | 19.9(11.5-32.2) | 14.5(11.1-21.5) | 19.5(10.2-39.3) |
| DBIL; umol/L | 12.1(6.5-31.2) | 9.4(5.5-25.3) | 9.0(4.9-18.9) | 6.6(3.4-9.8) | 9.2(3.7-21.1) |
| LN (%) | 2(5.9) | 0(0.0) | 0(0.0) | 0(0.0) | 1(3.4) |
| CEAID (%) | 1(2.9) | 1(5.6) | 2(14.3) | 0(0.0) | 1(3.4) |
| FA (%) | 2(5.9) | 0(0.0) | 0(0.0) | 0(0.0) | 0(0.0) |

Categorical variables were described as numbers (percentages) and continuous ones as median (range).

Abbreviations: NAFLD, non-alcoholic fatty liver disease; TP, total protein; ALB, albumin; GLB, globulin; ALT, alanine aminotransferase; AST, aspartate aminotransferase; ALP, alkaline phosphatase; GGT, γ-glutamyltransferase; TBIL, total bilirubin; DBIL, direct bilirubin; LN, abdominal lymph node enlargement (B-mode ultrasound); CEAID, current extrahepatic autoimmune diseases; FA, familial autoimmunity.

Supplementary Table 2: Baseline demographic and clinical characteristics of AILD patients.

| Features | AIH  (n=173) | PBC  (n=330) | PBC-AIH OS  (n=78) | All patients  (n=581) |
| --- | --- | --- | --- | --- |
| Gender (female/male) | 152/21 | 281/49 | 68/10 | 501/80 |
| Age males; y(range) | 56(52-63) | 62(56-71) | 52(50-67) | 60(50-70) |
| Age females; y(range) | 59(52-65) | 60(54-65) | 58(52-65) | 59(52-65) |
| Cirrhosis at initial, n (%) | 67(38.7) | 99(30.0) | 24(30.8) | 190(32.7) |
| Biochemical Parameters |  |  |  |  |
| TP; g/L | 77(71-82) | 76(69-81) | 76(69-84) | 76(69-82) |
| ALB; g/L | 38(33-43) | 39(33-43) | 38(34-42) | 39(34-43) |
| GLB; g/L | 38(33-42) | 35(31-41) | 39(34-46) | 37(32-42) |
| ALT; IU/l | 71(40-303) | 66(39-102) | 112(45-288) | 73(41-159) |
| AST; IU/l | 86(48-261) | 69(46-92) | 118(68-255) | 76.9(47-145) |
| ALP; IU/l | 153(100-239) | 210(127-349) | 204(124-356) | 179(115-328) |
| GGT; IU/l | 147(74-283) | 218(100-384) | 231(152-438) | 191(98-370) |
| TBIL; umol/L | 21(13.7-58.4) | 18.1(12.0-32.0) | 22.8(12.5-69.1) | 19.6(12.8-42.5) |
| DBIL; umol/L | 8.5(4.6-22.3) | 6.6(4.1-14.7) | 8.9(4.6-40.0) | 7.6(4.4-22.3) |
| Immunological Examinations |  |  |  |  |
| IgG (mg/dL) | 1910(1050-3590) | 1530(461-2830) | 1920(903-6360) | 1750(461-6360) |
| IgM (mg/dL) | 172(27-969) | 353(35-1710) | 318(42-1460) | 285(27-1710) |
| ANA (%) | 168(97.1) | 316(95.8) | 75(96.2) | 559(96.2) |
| AMA (%) | 3(1.7) | 289(87.6) | 56(71.8) | 348(59.9) |
| AMA-M2 (%) | 2(1.2) | 112(33.9) | 29(37.2) | 143(24.6) |
| Anti-SMA (%) | 35(20.2) | 35(10.6) | 17(21.8) | 87(15.0) |
| Anti-SLA/LP (%) | 2(1.2) | 0(0.0) | 0(0.0) | 2(0.3) |
| Anti-LKM1(%) | 4(2.3) | 0(0.0) | 0(0.0) | 4(0.7) |
| Anti-LC1(%) | 2(1.2) | 0(0.0) | 0(0.0) | 2(0.3) |
| Anti-Ro52/SSA (%) | 30(17.3) | 50(15.1) | 11(14.1) | 91(15.7) |
| Anti-Ro/SSA (%) | 26(15.0) | 30(9.1) | 8(10.3) | 64(11.0) |
| Anti-gp210 (%) | 5(2.9) | 42(12.7) | 9(11.5) | 56(9.6) |
| Anti-sp100 (%) | 9(5.2) | 38(11.5) | 6(7.7) | 53(9.1) |
| LN (%) | 66(38.2) | 129(39.1) | 31(39.7) | 226(38.9) |
| CEAID (%) | 71(41.0) | 149(45.2) | 37(47.4) | 257(44.2) |
| FA (%) | 24(13.9) | 19(5.8) | 7(9.0) | 50(8.6) |

Categorical variables were described as numbers (percentages) and continuous ones as median (range).

Detection of ANA, AMA, Anti-SMA, Anti-LKM: The tissue sections from rat liver, kidney, and stomach or Hep-2 tumor cells were stained with florecein-conjugated goat anti–human Ig and viewed in a fluorescence microscope, a titer ≥80 was considered significant. Detection of Anti-SLA/LP: ELISA.

Abbreviations: TP, total protein; ALB, albumin; GLB, globulin; ALT, alanine aminotransferase; AST, aspartate aminotransferase; ALP, alkaline phosphatase; GGT, γ-glutamyltransferase; TBIL, total bilirubin; DBIL, direct bilirubin; IgG, immunoglobulin G; IgM, immunoglobulin ; ANA, antinuclear antibody; AMA, anti-mitochondrial antibody; AMA-M2, anti-mitochondrial antibody-M2; SMA, anti-smooth muscle antibody; SMA, smooth muscle antibody; SLA, soluble liver antigen; LP, liver pancreas; LKM1, liver kidney microsomal type 1; LC1, liver cytosol type 1; gp210, anti-glucoprotein 210; LN, abdominal lymph node enlargement (B-mode ultrasound); CEAID, current extrahepatic autoimmune diseases; FA, familial autoimmunity.

Supplementary Table 3: The percentage of CEAID from the study group.

| Disease | AIH  (n=173) | PBC  (n=330) | PBC-AIH OS  (n=78) | All patients  (n=581) | HC  (n=782) |
| --- | --- | --- | --- | --- | --- |
| Combined with other autoimmune diseases | 71(41.0) | 149(45.2) | 37(47.4) | 257(44.2) | 39(5.0) |
| Autoimmune thyroid disease (%) | 28(16.2) | 24(7.3) | 16(20.5) | 68(11.7) | 19(2.4) |
| Sjögren’s syndrome (%) | 13(7.5) | 40(12.1) | 5(6.4) | 58(10.0) | 2(0.3) |
| Rheumatoid arthritis (%) | 12(6.9) | 50(15.2) | 7(9.0) | 69(11.9) | 14(1.8) |
| Systemic sclerosis (%) | 6(3.5) | 19(5.8) | 2(2.6) | 27(4.6) | 1(0.1) |
| Systemic lupus erythematosus (%) | 4(2.3) | 6(1.8) | 2(2.6) | 12(2.1) | 2(0.3) |
| Rheumatic heart disease (%) | 1(0.6) | 0(0.0) | 0(0.0) | 1(0.2) | 0(0.0) |
| Combined with 2 diseases (%) | 5(2.9) | 10(3.0) | 5(6.4) | 20(3.4) | 1(0.1) |
| Combined with 3 or more diseases (%) | 2(1.2) | 0(0.0) | 0(0.0) | 2(0.3) | 0(0.0) |
| None (%) | 102(59.0) | 181(54.8) | 41(52.6) | 324(55.8) | 743(95.0) |

Categorical variables were described as numbers (percentages).

Supplementary Table 4: Correlation matrix for the comparison between each predictive variable (n=581).

|  | GEN | AGE | TP | ALB | GLB | ALT | AST | ALP | GGT | TBIL | DBIL | LN | CEAID | | FA |
| --- | --- | --- | --- | --- | --- | --- | --- | --- | --- | --- | --- | --- | --- | --- | --- |
| GEN correlation coefficient  P value | 1 | 0.01 0.83 | -0.02 0.73 | 0.07 0.17 | -0.11 0.03 | -0.08 0.08 | -0.03 0.53 | -0.02 0.71 | -0.03 0.52 | -0.08 0.10 | -0.09 0.07 | -0.10 0.05 | 0.05  0.30 | -0.04 0.43 | |
| AGE correlation coefficient  P value |  | 1 | 0.05 0.27 | 0.00 0.96 | 0.05 0.32 | 0.02 0.70 | 0.00 0.96 | 0.10 0.04 | 0.08 0.09 | 0.13 0.01 | 0.18 0.00 | 0.06 0.25 | 0.05  0.27 | -0.03 0.54 | |
| TP correlation coefficient  P value |  |  | 1 | 0.36 0.00 | 0.62 0.00 | -0.04 0.39 | -0.09 0.06 | 0.14 0.00 | 0.17 0.00 | -0.18 0.00 | -0.16 0.00 | -0.06 0.20 | 0.03  0.54 | 0.04 0.43 | |
| ALB correlation coefficient  P value |  |  |  | 1 | 0.01 0.84 | -0.01 0.91 | -0.06 0.20 | -0.01 0.90 | 0.07 0.14 | -0.13 0.01 | -0.16 0.00 | -0.15 0.00 | 0.07  0.15 | 0.10 0.04 | |
| GLB correlation coefficient P value |  |  |  |  | 1 | 0.01 0.77 | 0.00 0.96 | 0.20 0.00 | 0.16 0.00 | -0.04 0.37 | -0.05 0.32 | 0.08 0.10 | 0.01  0.82 | -0.01 0.70 | |
| ALT correlation coefficient  P value |  |  |  |  |  | 1 | 0.73 0.00 | 0.01 0.82 | 0.04 0.45 | 0.37 0.00 | 0.35 0.00 | 0.19 0.00 | -0.03 0.55 | 0.02 0.66 | |
| AST correlation coefficient  P value |  |  |  |  |  |  | 1 | 0.00 0.97 | 0.03 0.59 | 0.40 0.00 | 0.41 0.00 | 0.18 0.00 | -0.04 0.36 | 0.01 0.77 | |
| ALP correlation coefficient  P value |  |  |  |  |  |  |  | 1 | 0.66 0.00 | 0.16 0.00 | 0.14 0.00 | 0.04 0.42 | -0.03 0.53 | -0.05 0.33 | |
| GGT correlation coefficient P value |  |  |  |  |  |  |  |  | 1 | 0.11 0.02 | 0.10 0.04 | 0.02 0.75 | -0.08 0.09 | -0.04 0.47 | |
| TBIL correlation coefficient  P value |  |  |  |  |  |  |  |  |  | 1 | 0.95 0.00 | 0.39 0.00 | -0.12 0.015 | -0.02 0.76 | |
| DBIL correlation coefficient  P value |  |  |  |  |  |  |  |  |  |  | 1 | 0.37 0.00 | -0.11 0.02 | -0.02 0.69 | |
| LN correlation coefficient  P value |  |  |  |  |  |  |  |  |  |  |  | 1 | -0.01 0.88 | -0.01 0.81 | |
| CEAID correlation coefficient P value |  |  |  |  |  |  |  |  |  |  |  |  | 1 | 0.33 0.00 | |
| FA correlation coefficient  P value |  |  |  |  |  |  |  |  |  |  |  |  |  | 1 | |

Notes: Correlations between variables were evaluated by Spearman’s rho coefficient. Decimal fractions were rounded to two places after the decimal point.

Abbreviations: TP, total protein; ALB, albumin; GLB, globulin; ALT, alanine aminotransferase; AST, aspartate aminotransferase; ALP, alkaline phosphatase; GGT, gamma-glutamyl transpeptidase; TBIL, total bilirubin; DBIL, direct bilirubin; LN, abdominal lymph node enlargement (B-mode ultrasound); CEAID, current extrahepatic autoimmune diseases; FA, familial autoimmunity.

Supplementary Table 5: Baseline demographic and clinical characteristics of cirrhosis patients and non-cirrhosis patients.

| Features | Cirrhosis patients  (n=191) | Non-cirrhosis patients  (n=390) | Statistic value | P value |
| --- | --- | --- | --- | --- |
| Gender(female/male) | 158/33 | 348/42 | 4.82 | 0.032 |
| Age, y(range) | 61(16-86) | 54(16-82) | 4.82 | <0.001 |
| Biochemical Parameters |  |  |  |  |
| TP (g/L) | 69(61-77) | 76(67-81) | 3.82 | <0.001 |
| ALB (g/L) | 35(30-40) | 42(35-45) | 6.45 | <0.001 |
| GLB (g/L) | 33(29-41) | 34(27-39) | 0.42 | 0.675 |
| ALT (IU/l) | 44(25-86) | 90(47-245) | 6.35 | <0.001 |
| AST (IU/l) | 56(33-104) | 75(46-170) | 3.39 | 0.001 |
| ALP (IU/l) | 153(93-262) | 148(96-258) | 0.76 | 0.866 |
| GGT (IU/l) | 133(51-291) | 166(84-331) | 0.95 | 0.017 |
| TBIL (µmol/L) | 22.5(13.3-40.1) | 17.0(10.7-33.5) | 0.94 | 0.019 |
| DBIL (µmol/L) | 8.2(5.2-23.1) | 6.2(3.9-16.4) | 1.14 | 0.015 |
| LN, (%) | 81(42.4) | 143(36.7) | 1.98 | 0.162 |
| CEAID, (%) | 97(50.8) | 157(40.3) | 6.01 | 0.016 |
| FA, (%) | 20(10.5) | 29(7.4) | 1.47 | 0.227 |

*Categorical variables were described as numbers (percentages) and continuous ones as median (range).

Abbreviations: TP, total protein; ALB, albumin; GLB, globulin; ALT, alanine aminotransferase; AST, aspartate aminotransferase; ALP, alkaline phosphatase; GGT, γ-glutamyltransferase; TBIL, total bilirubin; DBIL, direct bilirubin; LN, abdominal lymph node enlargement (B-mode ultrasound); CEAID, current extrahepatic autoimmune diseases; FA, familial autoimmunity.
