# Supplementary figures and images for: Predicting Liver Disease Risk Using a Combination of Common Clinical Markers: A Screening Model from Routine Health Check-Up
Source: Dis Markers. 2020 May 31;2020:8460883. doi: 10.1155/2020/8460883 (PMC7281844; doi:10.1155/2020/8460883)

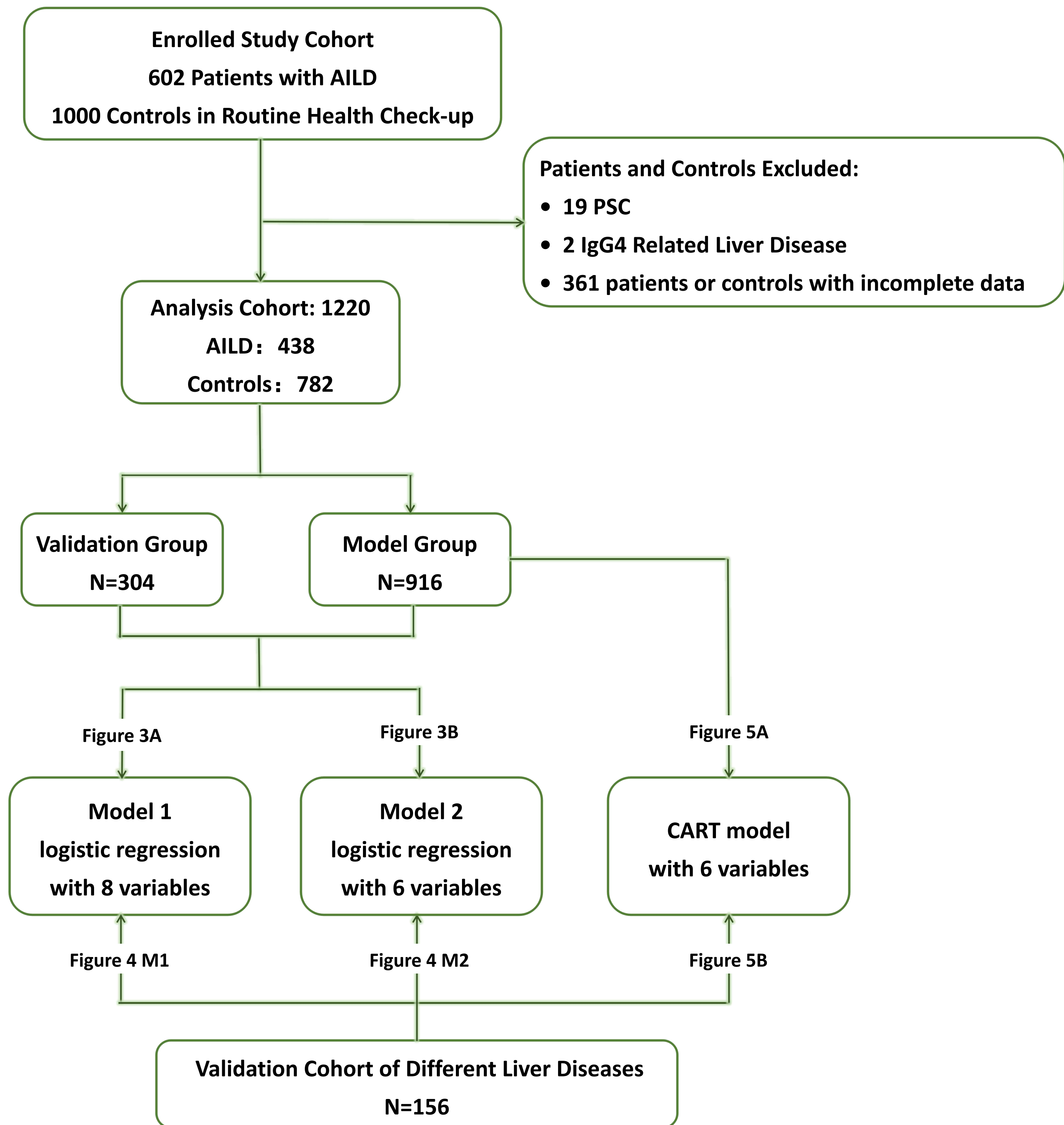

A

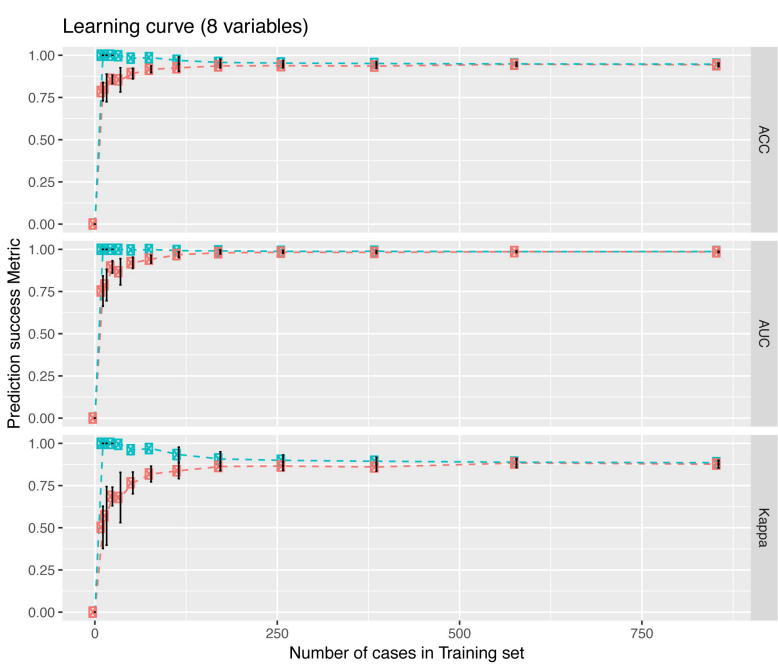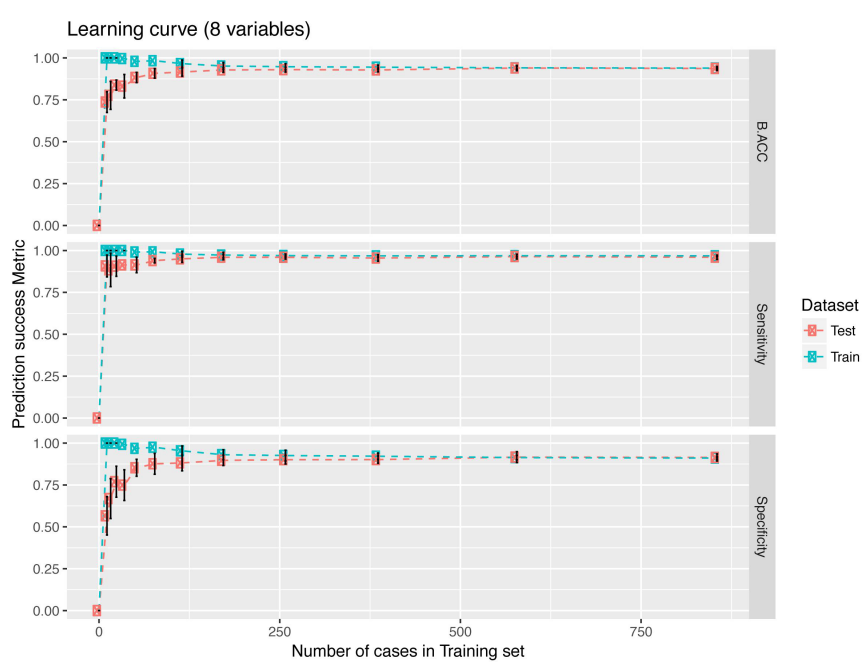

B

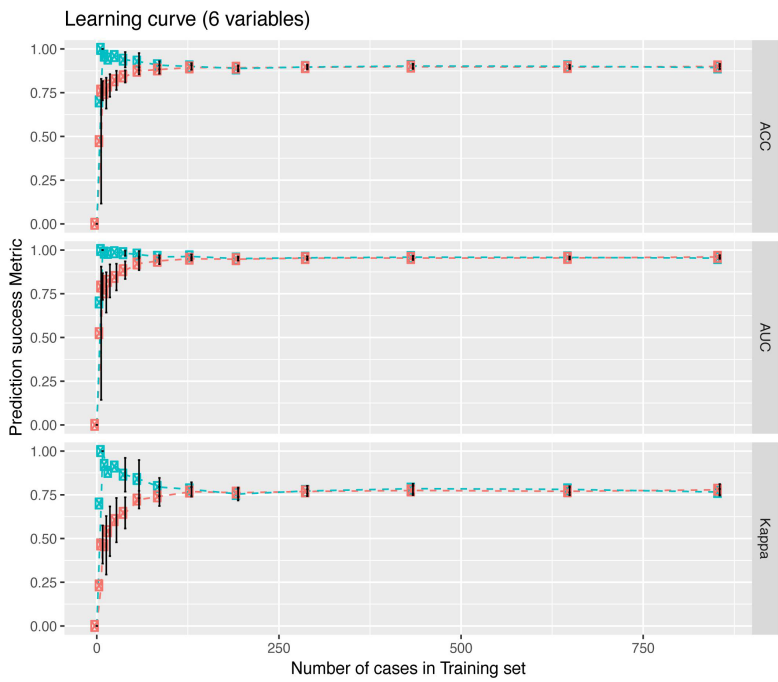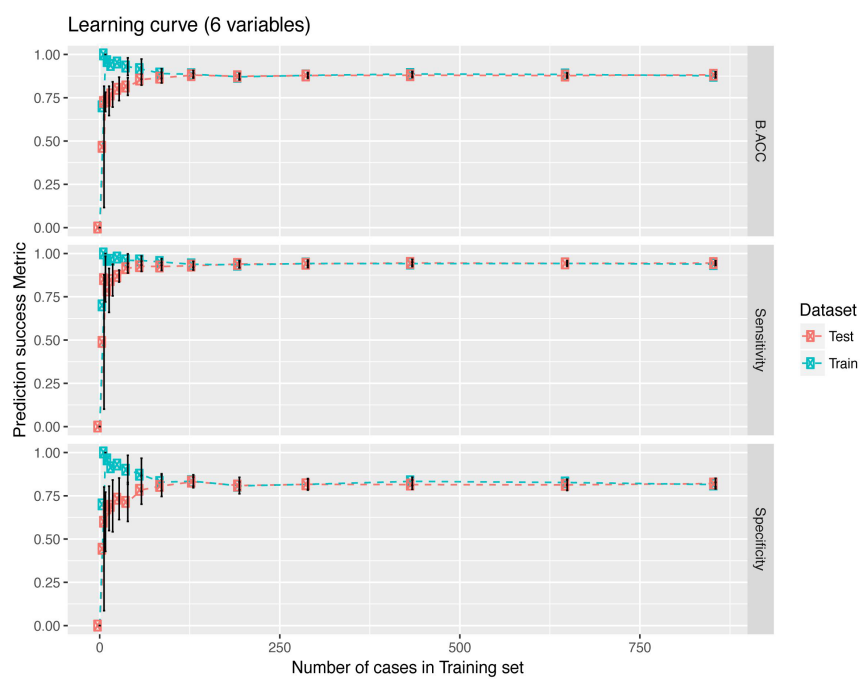

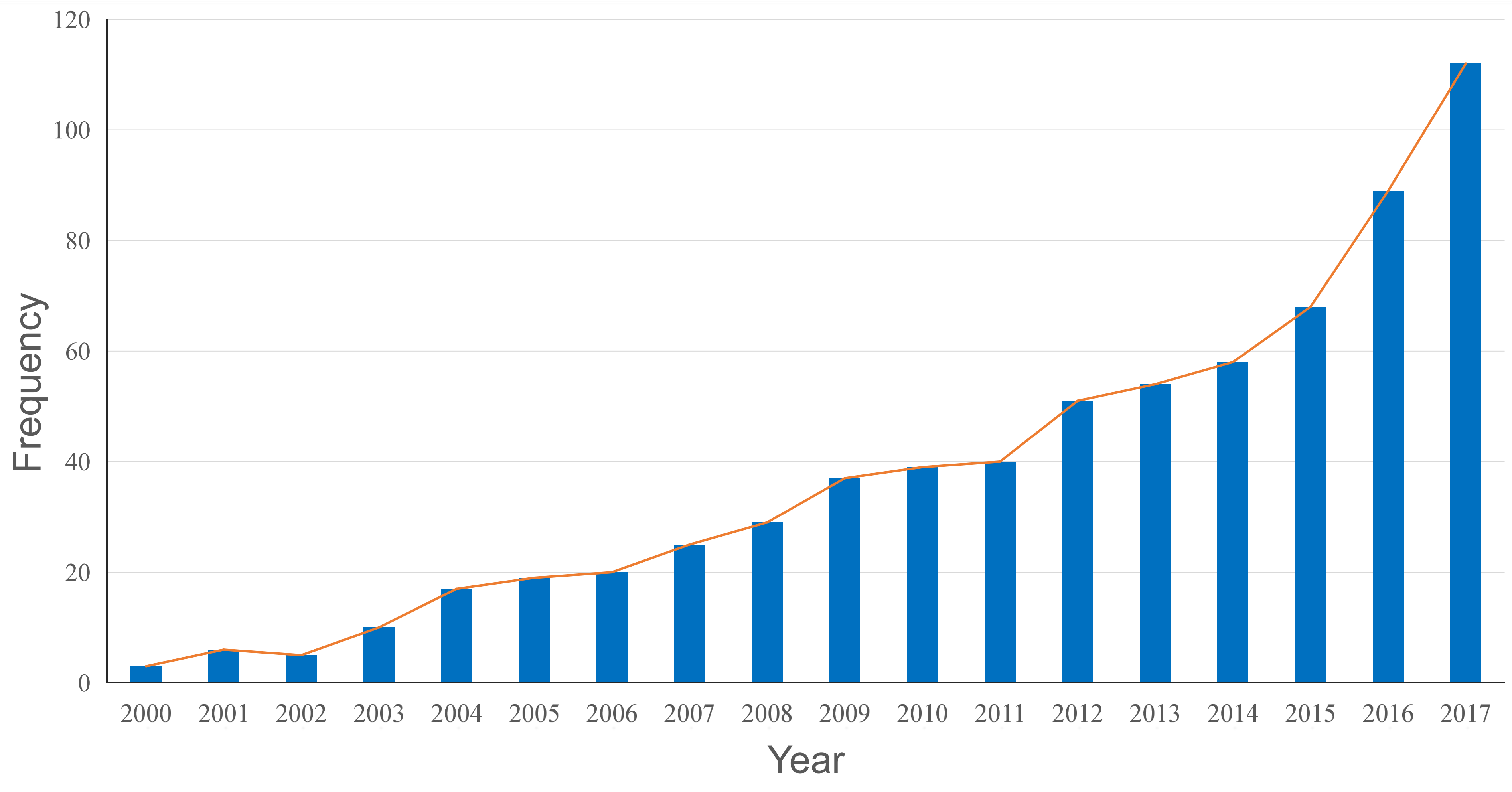

Supplement: Supplementary Materials — Supplementary Table 1: baseline demographic and clinical characteristics of other hepatic disease patients. Supplementary Table 2: baseline demographic and clinical characteristics of AILD patients. Supplementary Table 3: the percentage of CEAID from the study group. Supplementary Table 4: correlation matrix for the comparison between each predictive variable (n = 581). Supplementary Table 5: baseline demographic and clinical characteristics of cirrhosis patients and noncirrhosis patients. Supplementary Figure 1: flowchart of the inclusion criteria. Supplementary Figure 2: learning curve of regression model1 and model2. Supplementary Figure 3: the trend of newly diagnosed AILD patients yearly. [file 8460883.f1.zip › Supplement_Figures_new.pdf]
