# Supplementary material for: Predicting Liver Disease Risk Using a Combination of Common Clinical Markers: A Screening Model from Routine Health Check-Up
Source: Dis Markers. 2020 May 31;2020:8460883. doi: 10.1155/2020/8460883 (PMC7281844; doi:10.1155/2020/8460883)
Supplement: Supplementary Materials — Supplementary Table 1: baseline demographic and clinical characteristics of other hepatic disease patients. Supplementary Table 2: baseline demographic and clinical characteristics of AILD patients. Supplementary Table 3: the percentage of CEAID from the study group. Supplementary Table 4: correlation matrix for the comparison between each predictive variable (n = 581). Supplementary Table 5: baseline demographic and clinical characteristics of cirrhosis patients and noncirrhosis patients. Supplementary Figure 1: flowchart of the inclusion criteria. Supplementary Figure 2: learning curve of regression model1 and model2. Supplementary Figure 3: the trend of newly diagnosed AILD patients yearly. [file 8460883.f1.zip › Supplementary Materials_new.doc]

**Supplementary Materials**

**Participants**

Diagnostic criteria of AIH applied followed the descriptive diagnostic criteria updated in 1999^1^, patients were included if meeting the "Probable AIH". PBC was diagnosed with two of the three criteria satisfied^2,3^. In addition, the “Paris Criteria” was employed to diagnose PBC-AIH OS^4^. Control subjects were selected from individuals undergoing health check-up in 2017 at the same medical center, who were all confirmed without AILD or other liver injuries.

**Choosing Variables**

Multiple comparison was performed to select the most representative indicators. Female preponderance and age correlation were the feature of population studies of AILD, as almost universally admitted^5,6^. As for biochemical markers, immunoglobulins are the main GLB components, increased γ-globulin or immunoglobulin G (IgG) levels in AIH and higher immunoglobulin M (IgM) levels in PBC make GLB the main index in AILD. While assessing LFTs, strong correlations were found in the AILD cohort. Among them, ALT and GGT were selected for relatively appropriate sensitivity and specificity in early stages based on reported literature^7–9^, and in our clinical practice we consider ALT and GGT to be more informative than ALT and GGT for early detection of AILD.

With regard to the remaining factors, imaging studies showed that lymph node enlargement in the abdominal area was a typical ultrasound feature in AILD and other liver diseases^10,11^. In addition, the presence of extrahepatic autoimmune diseases was well recognized in AILD and reported frequently^12,13^. Besides, autoimmunity was common in the families of AILD patients, with an estimated 14–20% of first-degree relatives of PBC probands having an autoimmune disease other than PBC^14^. Approximately 42% of AIH patients reported a first-degree family member with another autoimmune disease^15^. Finally, we selected age, gender, GLB, ALT, GGT, LN, CEAIDs and FA as the most potential predictors for further regression analysis.

**Statistical Analysis**

***Descriptive analyses***

Categorical variables were described as frequencies (n) and percentages (%), and numerical ones as mean (95% confidence interval [CI]) or median (interquartile range). All analyses were performed at a significance level of 5% and the associated confidence intervals (CIs) were estimated for each relevant parameter. The Chi-square test for trend was applied to assess disease rate changes. Categorical and numerical variables were respectively analyzed by nonparametric Chi-squared test (two-tailed) and Mann-Whitney U test, as appropriate.

Considering all controls are from year 2017, each relevant clinical variable was tested for potential confounding effect of the year for sampling. Samples were grouped by year and compared to samples from other years using Wilcoxon Rank Sum Test for numerical variables (age, ALT, GGT, GLB) and Fisher’s exact test for categorical variables (probability of female, LN, FA and CEAID). No significant differences were observed at test P value 0.05 (adjusted for multiple testing via FDR correction).

***Classification and Regression Tree***

CART searches for combinations of values of independent variables that best predict the diagnosis of AILD. Results from CART were presented as “decision trees” that required no calculation for their use. It relied on statistically optimum recursive splitting of the patients into smaller and smaller subgroups, based on the critical levels of the predictive variables^16^.

Each splitting node gives rise to further nodes, one to the left and one to the right. These may in turn become further splitting nodes or else may be terminal nodes. Each terminal node is allocated a class, usually the class most strongly represented at that node. In this way, given the whole tree, every trial is uniquely allocated a class. The tree and its decision rules constitute the model^17^.

Briefly, this method allows the construction of inductive decision trees through strict binary splitting. There are no specific splitters were forced for root or child nodes. This algorithm divides each parent node into two homogeneous child nodes by applying yes/no answers at each decision node. Cut-off points for continuous and categorical variables were automatically generated by the model, based on statistical cost assumptions. The basic tree-generating process consists of: 1) selecting the most discriminative variable according to an impurity function to partition the data; 2) repeating this partition until child nodes are considered pure enough to be terminal and 3) pruning the resulting tree to avoid overfitting and redundancy^18,19^.

In our study, the tree presented was the one that minimizes the overall cross-validated relative error estimate, that which most accurately predicts data excluded from forming the tree. All in all, CART methods were used to determine which common health check-up items best predicted early detection on AILD.

**References**

1.	Alvarez F, Berg PA, Bianchi FB, et al. International Autoimmune Hepatitis Group Report: Review of criteria for diagnosis of autoimmune hepatitis. *J Hepatol*. 1999. doi:10.1016/S0168-8278(99)80297-9

2.	EJ C, AH A, Review LKDPT-J. Primary biliary cirrhosis MISC1 - DD:20151123. *Lancet (london, england)*. 2015. doi:10.1016/S0140-6736%2815%2900154-3 DBP - EM:201548 TN - NCT02135536

3.	European Association for the Study of the Liver. EASL Clinical Practice Guidelines: Management of cholestatic liver diseases. *J Hepatol*. 2009. doi:10.1016/j.jhep.2009.04.009

4.	Trivedi PJ, Hirschfield GM. Review article: Overlap syndromes and autoimmune liver disease. *Aliment Pharmacol Ther*. 2012. doi:10.1111/j.1365-2036.2012.05223.x

5.	Lee YM, Teo EK, Ng TM, Khor C, Fock KM. Autoimmune hepatitis in Singapore: A rare syndrome affecting middle-aged women. *J Gastroenterol Hepatol*. 2001. doi:10.1046/j.1440-1746.2001.02646.x

6.	Cookson S, Constantini PK, Clare M, et al. Frequency and nature of cytokine gene polymorphisms in type 1 autoimmune hepatitis. *Hepatology*. 1999. doi:10.1002/hep.510300412

7.	Van Der Poorten D, Kenny DT, Butler T, George J. Liver disease in adolescents: A cohort study of high-risk individuals. *Hepatology*. 2007. doi:10.1002/hep.21918

8.	Nevens F, Andreone P, Mazzella G, et al. A Placebo-controlled trial of obeticholic acid in primary biliary cholangitis. In: *New England Journal of Medicine*. ; 2016. doi:10.1056/NEJMoa1509840

9.	Robles-Diaz M, Garcia-Cortes M, Medina-Caliz I, et al. The value of serum aspartate aminotransferase and gamma-glutamyl transpetidase as biomarkers in hepatotoxicity. *Liver Int*. 2015. doi:10.1111/liv.12834

10.	Malik N, Venkatesh SK. Imaging of autoimmune hepatitis and overlap syndromes. *Abdom Radiol*. 2017. doi:10.1007/s00261-016-1019-x

11.	Dietrich CF, Leuschner MS, Zeuzem S, et al. Peri-hepatic lymphadenopathy in primary biliary cirrhosis reflects progression of the disease. *Eur J Gastroenterol Hepatol*. 1999. doi:10.1097/00042737-199907000-00012

12.	Wong GW, Yeong T, Lawrence D, Yeoman AD, Verma S, Heneghan MA. Concurrent extrahepatic autoimmunity in autoimmune hepatitis: implications for diagnosis, clinical course and long-term outcomes. *Liver Int*. 2017. doi:10.1111/liv.13236

13.	Efe C, Wahlin S, Ozaslan E, et al. Autoimmune hepatitis/primary biliary cirrhosis overlap syndrome and associated extrahepatic autoimmune diseases. *Eur J Gastroenterol Hepatol*. 2012. doi:10.1097/MEG.0b013e328350f95b

14.	Watt FE, James OFW, Jones DEJ. Patterns of autoimmunity in primary biliary cirrhosis patients and their families: A population-based cohort study. *QJM - Mon J Assoc Physicians*. 2004. doi:10.1093/qjmed/hch078

15.	Van Gerven NMF, Verwer BJ, Witte BI, et al. Epidemiology and clinical characteristics of autoimmune hepatitis in the Netherlands. *Scand J Gastroenterol*. 2014. doi:10.3109/00365521.2014.946083

16.	Rovlias A, Kotsou S. Classification and regression tree for prediction of outcome after severe head injury using simple clinical and laboratory variables. *J Neurotrauma*. 2004. doi:10.1089/0897715041526249

17.	Evans DG, Everis LK, Betts GD. Use of survival analysis and Classification and Regression Trees to model the growth/no growth boundary of spoilage yeasts as affected by alcohol, pH, sucrose, sorbate and temperature. *Int J Food Microbiol*. 2004. doi:10.1016/j.ijfoodmicro.2003.07.008

18.	Altamirano J, López-Pelayo H, Michelena J, et al. Alcohol abstinence in patients surviving an episode of alcoholic hepatitis: Prediction and impact on long-term survival. *Hepatology*. 2017. doi:10.1002/hep.29338

19.	Mckinney WP, Young MJ, Hartz A, Fong Lee M. The Inexact Use of Fisher’s Exact Test in Six Major Medical Journals. *JAMA J Am Med Assoc*. 1989. doi:10.1001/jama.1989.03420230084031

**Figure Legends**

**Supplementary Figure 1:** Flowchart of the inclusion criteria.

A total of 602 AILD patients and 1000 controls which were randomly collected from routine health check-up in past 18 years. Within the individuals, 19 PSC, 2 IgG4 related liver disease and 361 individuals with incomplete data were excluded. As a result, 438 patients and 782 controls were used to establish and verify the prediction model. In order to validate the regression model 2, another cohort with abnormal LFTs (including 56 AILD and 100 other liver diseases patients) were further collected. AILD, autoimmune liver disease.

**Supplementary Figure 2:** Learning curve of regression model1 and model2.

Cross validation was used in training and test set. The x-axis is the number of cases. The y-axis shows accuracy (ACC), the area under curve (AUC), Kappa and balanced accuracy (B.ACC), sensitivity, specificity in the left and right graphs of training and test set, respectively (with bootstrap 500 times).

**Supplementary Figure 3:** The trend of newly diagnosed AILD patients yearly.

From year 2001 to 2017, the diagnosis rate of AILD patients increased year by year (from 3 cases in 2001 to 83 cases in 2017).
